# Prenatal Protection: Maternal Diet May Modify Impact of PAHs

**DOI:** 10.1289/ehp.121-A311

**Published:** 2013-10-01

**Authors:** Julia R. Barrett

**Affiliations:** Julia R. Barrett, MS, ELS, a Madison, WI–based science writer and editor, has written for *EHP* since 1996. She is a member of the National Association of Science Writers and the Board of Editors in the Life Sciences.

Prenatal exposure to polycyclic aromatic hydrocarbons (PAHs) has previously been associated with higher incidence of depression, anxiety, and attention problems among highly exposed children.^1^^,^^2^ Although epidemiological studies have found clear associations between PAH exposure and adverse effects on fetal growth,^3^^,^^4^ evidence for impacts on growth has been inconsistent in biomarker-based research.^5^^,^^6^ A new study describes an association between prenatal PAH exposure and reduced birth weight that may be modified by mothers’ fruit and vegetable intake during pregnancy.^7^

PAHs are produced by incomplete combustion of diverse organic materials, including fossil fuels, tobacco, and foods.^8^ Once PAHs are inhaled or ingested, they are metabolized to compounds that can readily attach to DNA, forming so-called bulky DNA adducts that can set the stage for mutation and carcinogenesis.^9^ Bulky DNA adducts can be measured in cord blood as an indicator of a newborn’s prenatal exposure to several genotoxic agents, including PAHs.^7^

In the current study, the researchers used data collected for 612 newborns through the Newborns and Genotoxic Exposure Risks (NewGeneris) project, a multicenter European study investigating how contaminants in the diets of pregnant women affect their children’s health.^10^ The women were enrolled from 2006 to 2010 at 11 maternity units in Denmark, England, Greece, Norway, and Spain, and provided personal information and completed detailed food questionnaires. Birth information, including weight and head circumference, was gathered from medical records. Cord blood collected at birth was analyzed for levels of bulky DNA adducts at three laboratories in Hungary, Sweden, and the Netherlands.^7^

**Figure 1:**
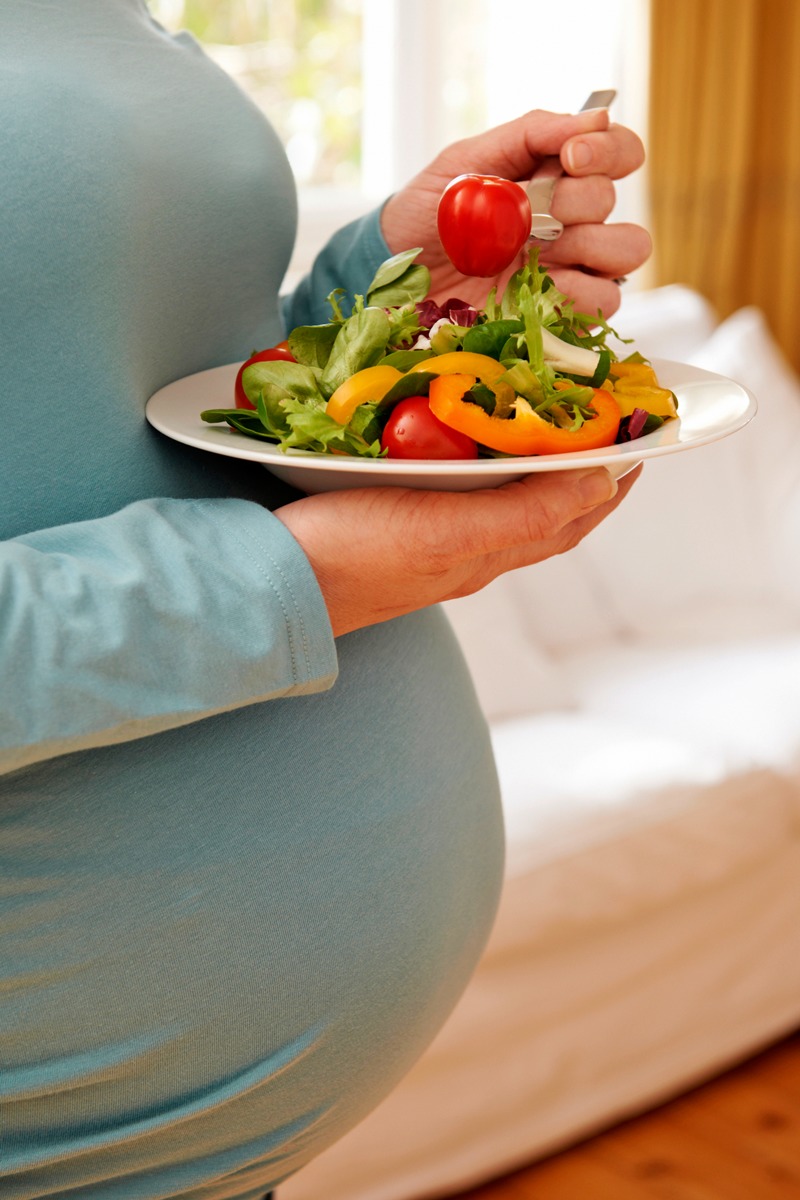
Mothers who eat a diet rich in fruits and vegetables may offer their unborn children some protection against adverse effects of PAHs. © MachineHeadz/Getty Images

Bulky DNA adducts are not a specific biomarker but a broad biomarker, says coauthor Marie Pedersen, a postdoctoral researcher at the Centre for Research in Environmental Epidemiology in Barcelona, Spain. They are therefore well suited for studying complex exposures such as diet and traffic emissions. “I think that’s a strength for this study,” Pedersen says.

The researchers categorized adduct levels as low, moderate, or high and considered several statistical models for various birth outcomes and numerous confounding factors. Analyses were conducted for the entire study population, by country, and by groupings of southern (Greece and Spain) versus northern (England, Denmark, and Norway) countries.

For the entire study population, newborns in the high-adduct group had an average birth weight of 129 g less than newborns in the low-adduct group. The southern countries had, on average, higher adduct levels and lower birth weights than the northern group, but high adduct levels were associated with high, not low, birth weight. (Pedersen says the adducts in the south may reflect different exposures than those in the north, or there may have been some protective factor involved in the southern pregnancies.) In women with low intakes of fruits and vegetables, there was a stronger association between high adduct levels and low birth weight (an estimated 248-g reduction for high versus low adduct levels) than there was among women with high fruit and vegetable intakes (an estimated 58-g decrease for high versus low adduct levels).

Radim J. Šrám, head of the Department of Genetic Ecotoxicology at the Institute of Experimental Medicine AS CR in Czech Republic, who was not involved with the study, says of his own research on PAHs and human health, “According to our experience, you get better information about the quality of fruits and vegetables intake when you measure vitamin C and E in the plasma, than when you calculate it according to the answers in questionnaires.” Pedersen and her colleagues indicate that further study is needed to test and possibly expand on their findings. “I’m already doing the analysis for the next study, and I can see that there are many modifiable factors that we can work on,” says Pedersen.
